# TGFβ Imprinting During Activation Promotes Natural Killer Cell Cytokine Hypersecretion

**DOI:** 10.3390/cancers10110423

**Published:** 2018-11-05

**Authors:** Jennifer A. Foltz, Jena E. Moseman, Aarohi Thakkar, Nitin Chakravarti, Dean A. Lee

**Affiliations:** 1 Nationwide Children’s Hospital, Center for Childhood Cancer and Blood Diseases, Columbus, OH 43205, USA; Jennifer.A.Foltz@wustl.edu (J.A.F.); Jena.Moseman@nationwidechildrens.org (J.E.M.); Aarohi.Thakkar@nationwidechildrens.org (A.T.); Nitin.Chakravarti@nationwidechildrens.org (N.C.); 2 Department of Pediatrics, The Ohio State University, Columbus, OH 43210, USA

**Keywords:** natural killer cells, TGFβ, IFNγ, cell therapy, IL-2, cytokines, immune therapy, tumor microenvironment

## Abstract

Transforming growth factor-beta (TGFβ) is a potent immunosuppressive cytokine that inhibits the anti-tumor responses of NK cells and T cells. However, the stimulation of natural killer (NK) cells with pro-inflammatory cytokines decreases NK cell sensitivity to TGFβ. Herein, we sought to determine if TGFβ imprinting (TGFβi) during NK cell activation and expansion would decrease NK cell sensitivity to TGFβ suppression. To this end, we demonstrate that the activation of NK cells during chronic IL-2 stimulation and TGFβi potently induces NK cell hypersecretion of interferon-gamma (IFNγ) and tumor necrosis factor-alpha (TNFα) in response to tumor targets which persists for at least one month in vitro after the removal of TGFβ. TGFβi NK cell cytokine hypersecretion is induced following both cytokine and tumor activation. Further, TGFβi NK cells have a marked suppression of SMAD3 and T-bet which is associated with altered chromatin accessibility. In contrast to their heightened cytokine secretion, TGFβi NK cells downregulate several activating receptors, granzyme and perforin, and upregulate TRAIL, leading to cell-line-specific alterations in cytotoxicity. These findings may impact our understanding of how TGFβ affects NK cell development and anti-tumor function.

## 1. Introduction

Natural Killer (NK) cells are part of the innate immune system and are critical in the immune surveillance of both virally infected cells and cancerous cells. NK cells can induce target cell death through multiple mechanisms including granzyme/perforin release, secretion of tumor necrosis factor-alpha (TNFα), and receptor-mediated cytotoxicity by TNF-related apoptosis inducing ligand (TRAIL) and FasL [1,2]. NK cells are activated to kill malignant cells using activating and inhibitory receptors that engage with stress and self-ligands expressed on malignant cells [3,4]. These receptors include the natural cytotoxicity receptors- NKp46, NKp44, and NKp30, along with NKG2D, DNAX Accessory Molecule-1 (DNAM-1), Fc gamma receptor III (CD16), and TRAIL, and the family of activating and inhibitory killer immunoglobulin receptors (KIR). NK cells are commonly distinguished by the intensity of their surface expression of CD56. CD56^bright^ NK cells largely lack the expression of CD16. Functionally, CD56^bright^ NK cells are potent cytokine producers, but are weakly cytotoxic unless pre-activated [5]. On the other hand, CD56^dim^ NK cells express high levels of CD16. CD56^dim^ NK cells are less potent cytokine producers, but are highly cytotoxic. Further, CD56^bright^ NK cells are predominantly located within tissues, whereas CD56^dim^ NK cells make up the majority of peripheral blood NK cells [6,7]. 

NK cell activation is regulated not only by the expression of ligands on target cells, but also by cytokine priming. NK cell function can be augmented by stimulation with interleukin (IL)-2, IL-12, IL-15, IL-18, and IL-21. IL-15 and IL-2 promote NK cell cytotoxicity, survival, and proliferation through the activation of STAT signaling, PI3K, and MAPK pathways [8,9]. IL-21, which primarily signals through STAT3, promotes NK cell proliferation and NKG2D expression [10,11]. Importantly, simultaneous stimulation of NK cells with multiple cytokines has distinct effects on NK cell function that differs from stimulation by the same cytokines separately. For example, simultaneous stimulation with IL-12, IL-15, and IL-18 generates cytokine-induced memory-like NK cells with significantly increased interferon-gamma (IFNγ) secretion following recall weeks later, whereas stimulation with IL-12, IL-15, or IL-18 individually does not generate cytokine-induced memory-like NK cells [12].

In addition, NK cell function is regulated by anti-inflammatory cytokines such as IL-10, IL-37, and transforming growth factor beta (TGFβ) [13,14]. TGFβ is a potent suppressor of the NK cell anti-tumor response and is present at elevated levels in the tumor microenvironment [15,16,17]. TGFβ activates SMAD2 and SMAD3 downstream of TGFβRI/II causing suppression of NK cell IFNγ production by downregulating the transcription factors T-bet and E4BP4 [18,19,20]. Further, TGFβ inhibits NK cell cytotoxicity by decreasing granzyme and perforin, and downregulates several activating receptors including NKG2D, NKp30, DNAM-1, TRAIL, and CD16, thereby inhibiting NK cell recognition of malignant cells expressing their cognate ligands [21,22,23,24,25,26,27,28,29]. Importantly, TGFβ has an acute effect on NK cell cytokine production and cytotoxicity, with the inhibition of function observed in primary NK cells following only a few hours of TGFβ exposure. The TGFβ suppression of NK cell function can be partially abrogated by the stimulation of NK cells with IL-12/IL-15/IL-18 or with ALT-803, an IL-15 superagonist/IL-15Rα Sushi-Fc fusion complex [19,30].

Since NK cells may be chronically exposed to TGFβ during tumor infiltration, we sought to determine if activation of NK cells in the presence of TGFβ such as in the tumor microenvironment would decrease NK cell sensitivity to TGFβ. To this end, we found that the activation of NK cells by either tumor stimuli or cytokines in the presence of TGFβ induces potent NK cell anti-tumor IFNγ and TNFα secretion and alterations in NK cell cytotoxicity, corresponding to the downregulation of T-bet and SMAD3 expression. 

## 2. Results

### 2.1. Chronic Stimulation by TGFβ during NK Cell Expansion and Activation Generates NK Cells with Increased Cytokine Secretion

**Figure 1:**
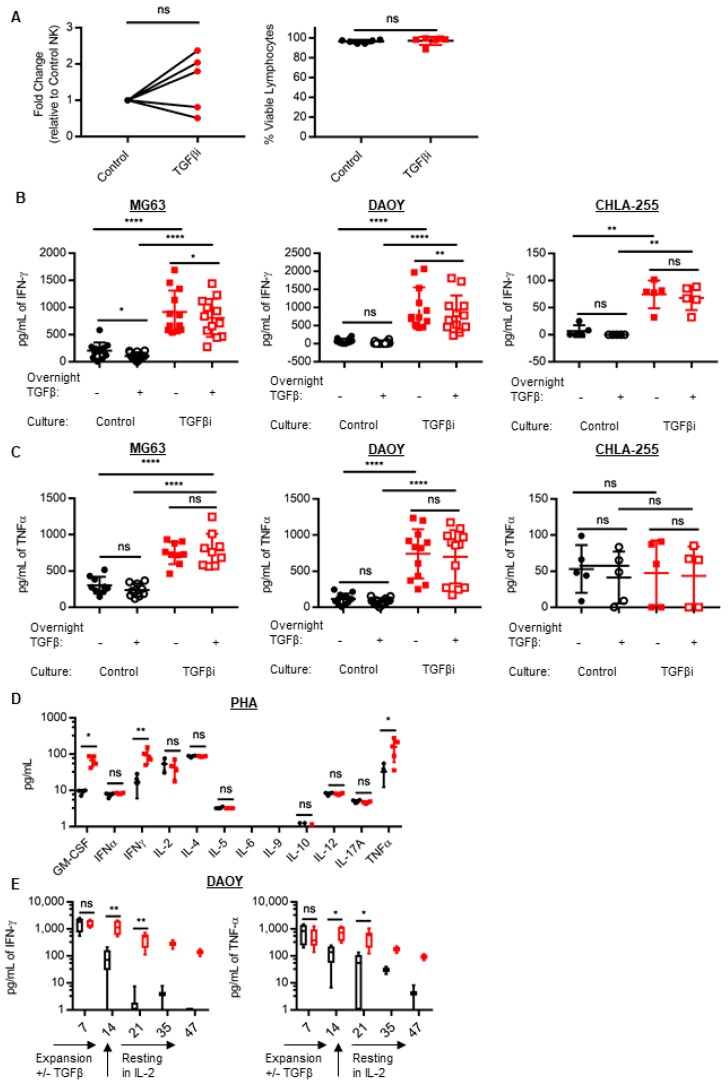
The chronic stimulation by transforming growth factor-beta (TGFβ) during natural killer (NK) cell expansion and activation generates NK cells with increased cytokine secretion. NK cells were cultured for 14 days in 50 IU/mL IL-2 or 50 IU/mL IL-2 plus 10 ng/mL TGFβ (TGFβi) with a weekly stimulation with K562 mbIL-21 feeder cells. (**A**) NK cell proliferation was compared by determining the fold change of control and TGFβi total NK (CD3^−^/CD56^+^ and CD3^−^/CD16^+^) cells at Day 14 and the viability of total cells was determined using Tonbo Viability dye. (**B**) After 14 days of expansion, NK cells were rested overnight with 50 IU/mL IL-2 (− TGFβ, baseline) or 50 IU/mL IL-2 plus 10 ng/mL TGFβ (+ TGFβ, acute TGFβ treatment). NK cells were then co-cultured with tumor targets for 3 h in fresh media (under identical cytokine conditions as used in the overnight rest) and supernatants were collected to measure interferon-gamma (IFNγ) and tumor necrosis factor-alpha (TNFα) cytokine secretion using Cytometric Bead Array analysis. Individual data points are depicted for MG63 (osteosarcoma) (IFNγ: *n* = 12, TNFα: *n* = 9), DAOY (medulloblastoma) (*n* = 12), and CHLA-255 (neuroblastoma) (*n* = 5). (**D**) The control and TGFβi NK cells were stimulated with 10 µg/mL of PHA at 2 e6 NK cells/mL for 4 h and cytokine secretion was measured by cytometric bead array (CBA) or a MACSPlex Cytokine 12 Kit. Individual data points depicted. Lines and bars represent Mean ± SD. (**E**) TGFβi and control NK cell anti-tumor cytokine secretion following overnight treatment in fresh media with 50 IU/mL IL-2 was assessed against DAOY at Day 7 and Day 14 of expansion, and after removal from expansion conditions at Day 21, 35 and 47 +/− 1 day as described for Figure 1**B**,**C**. (Day 7 *n* = 5, Day 14 and 21 *n* = 6, Day 35 and 47, *n* = 2)). Median with min to max whiskers depicted. Control in black, TGFβi in red. Statistical differences were determined by paired *t*-test (**A**,**D**,**E**) and two-way repeated measures ANOVA with Holm–Sidak’s multiple comparisons test for all others. * *p* ≤ 0.05, ** *p* ≤ 0.01, *** *p* ≤ 0.001, **** *p* ≤ 0.0001. See also Figures S1 and S2.

To investigate the effect of chronic TGFβ stimulation during NK cell propagation and activation, NK cells were isolated from healthy peripheral blood and TGFβ imprinted (TGFβi) for 14 days by culturing with both IL-2 and 10 ng/mL TGFβ (a concentration previously reported to activate TGFβ signaling and suppress NK cell function) in combination with a weekly stimulation with irradiated K562 feeder cells expressing membrane-bound IL-21 (mbIL21) and 4-1BBL, which are known to induce robust NK cell proliferation and activation, resulting in a median ~2,500-fold expansion in 14 days [11]. TGFβ had a minimal impact on K562mbIL-21.41BBL induced NK cell proliferation, with NK cell expansion ranging from 465 to 3200-fold and an average viability that was greater than 96% (Figure 1A).

Since TGFβ is a potent inhibitor of IFNγ and TNFα secretion, we next sought to determine cytokine secretion of donor-matched control and TGFβi NK cells at the end of the 14 days of expansion. NK cells were rested overnight without TGFβ (baseline) and after acute TGFβ treatment (rested overnight in TGFβ). TGFβi significantly increased IFNγ secretion against all tumor targets tested (Figure 1B), and significantly increased TNFα secretion against all tumor targets except CHLA-255 (Figure 1C). When TGFβ was included in the cytotoxicity assay, it significantly suppressed the IFNγ secretion of control NK cells against MG63, and of TGFβi NK cells against MG63 and DAOY, but not CHLA-255 (Figure 1B). However, CHLA-255 stimulated less cytokine secretion than DAOY and MG63 from both the control and TGFβi NK cells. Neither TGFβi NK nor control NK cell TNFα secretion was significantly inhibited by acute TGFβ treatment against any cell line tested (Figure 1C). Tumors cultured alone in IL-2 or IL-2 plus TGFβ did not produce any detectable IFNγ or TNFα.

Next, we wanted to determine if this effect was due to an increase in the percentage of cytokine-producing NK cells or an increase in the amount of cytokine produced by each NK cell. To this end, we found that TGFβi significantly increased the percentage of cytokine-producing NK cells in response to tumor targets (Figure S1). Further, of the cytokine-producing NK cells, there was an increased intensity of IFNγ and TNFα (gMFI) in TGFβi NK cells (Figure S2), suggesting that TGFβi increases both the percentage of NK cells secreting cytokine and the amount of cytokine produced by the NK cells.

To determine if TGFβi effected the secretion of cytokines other than IFNγ and TNFα irrespective of the tumor target, TGFβi and control NK cells were stimulated with phytohaemagglutinin (PHA) for 4 h. Following PHA stimulation, we found that TGFβi NK cells produced significantly more IFNγ and TNFα, and granulocyte-macrophage colony-stimulating factor (GM-CSF), but the TGFβi NK cells were not different from control NK cells in IFNα, IL-2, IL-4, IL-5, IL-10, IL-12, or IL-17A secretion. We were unable to detect any secretion of IL-6 or IL-9 in any of the donors tested (Figure 1D). Therefore, TGFβi selectively modifies NK cell cytokine secretion.

We next sought to determine the onset of TGFβi NK cell cytokine hypersecretion and the duration of cytokine hypersecretion following their removal from the imprinting conditions. NK cells were expanded for 14 days with K562mbIL-21.41BBL and subsequently removed from their expansion conditions and cultured in IL-2 alone. The secretion of IFNγ and TNFα by NK cells in response to tumor target stimulation (DAOY) following overnight treatment with IL-2 was measured in supernatants at Day 7, 14, and 1 week, 3 weeks, and 1 month (33 days) post-expansion. TGFβi NK cells demonstrated the onset of cytokine hypersecretion after 14 days of culture with K562.mbIL-21.41BBL and TGFβ (Figure 1E). Following removal from TGFβ, TGFβi NK cells maintained their significantly increased cytokine hypersecretion for 33 days following TGFβ stimulation, whereas the control NK cells exhibited a rapid decline in IFNγ secretion as early as day 21 of culture (1 week post-expansion). Thus, chronic stimulation with K562mbIL-21.41BBL feeder cells and TGFβ reprograms NK cells to become pro-inflammatory cytokine secretors that persist after the removal of IL-21 and TGFβ signals.

### 2.2. NK Cell Activation is Required for TGFβ Induced Cytokine Hypersecretion

**Figure 2:**
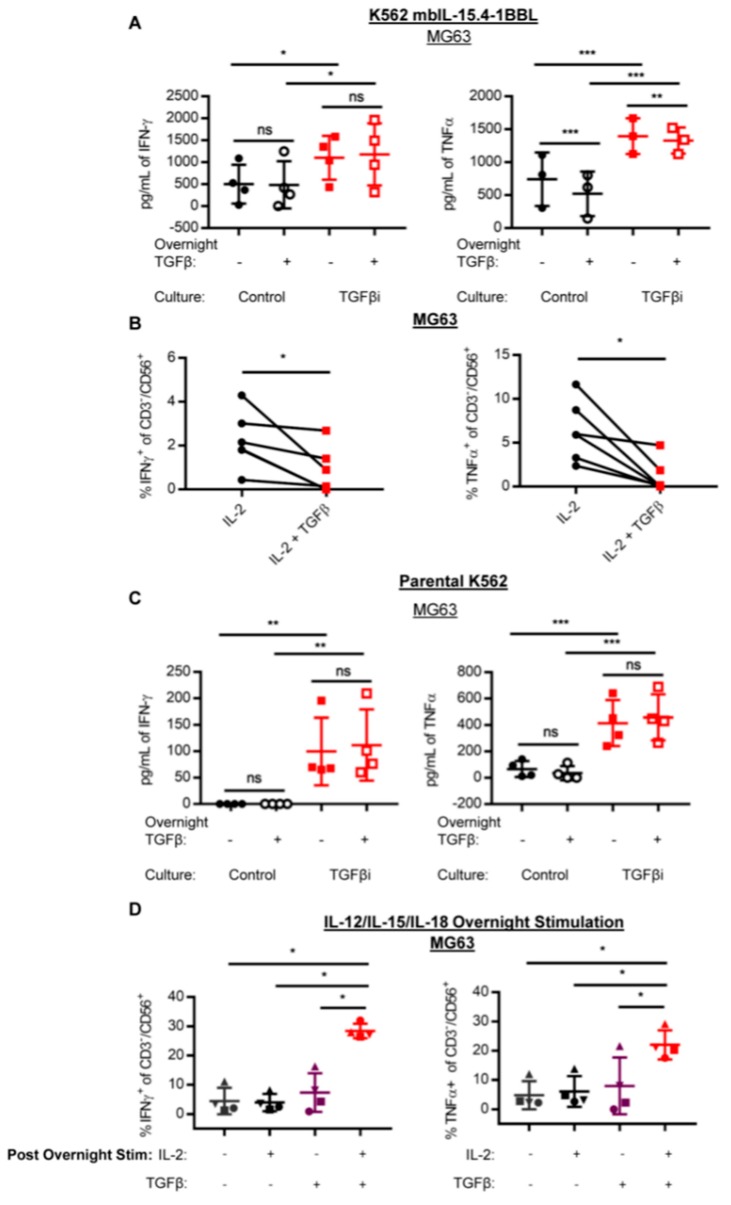
NK cell activation is required for TGFβ induced cytokine hypersecretion. NK cells were cultured with IL-2 alone or IL-2 plus 10 ng/mL TGFb and (**A**) K562mbIL-15 (IFNγ: *n* = 4, TNFα: *n* = 3) or (**B**) no feeder cell (*n* = 6), or (**C**) parental K562 (*n* = 4) for 7–14 days. Following culture, anti-tumor cytokine secretion or production by CBA or intracellular flow cytometry was assessed against an MG63 tumor target. (**D**) NK cells were stimulated overnight with 10 ng/mL IL-12, 50 ng/mL IL-15, and 50 ng/mL IL-18 plus or minus IL-2 and TGFβ. Following overnight stimulation, the NK cells were cultured with 1 ng/mL IL-15 plus or minus IL-2 and TGFβ. After 7–14 days of culture, anti-tumor IFNγ and TNFα production in response to MG63 was measured by intracellular flow cytometry (*n* = 4). Percent anti-tumor IFNγ+ and TNFα+ NK cells normalized to no target depicted for (B) and (D). Individual data points depicted for all. Lines and bars represent Mean ± SD. Statistical differences were determined by paired t-test for 2B and two-way repeated measures ANOVA with Holm–Sidak’s multiple comparisons test for all other graphs. * *p* ≤ 0.05, ** *p* ≤ 0.01, *** *p* ≤ 0.001, **** *p* ≤ 0.0001. See also Figure S3.

Since the feeder cells utilized for NK cell propagation in Figure 1 express membrane-bound IL-21 and IL-21 is known to modify TGFβ signaling in T-cells, we sought to determine if membrane-bound IL-21 is required for TGFβi by using K562 that express membrane-bound IL-15 instead of membrane-bound IL-21. Membrane-bound IL-15 expressing K562 also induced increased IFNγ and TNFα secretion by TGFβi NK cells at both the baseline and in the presence of acute overnight TGFβ treatment compared to the control NK cells (Figure 2A). Thus, membrane-bound IL-21 is not required for TGFβi. Under these conditions, TNFα secretion, but not IFNγ secretion, was inhibited by acute TGFβ treatment in both the control and TGFβi NK cells.

Next, we wanted to determine whether K562 feeder cells are required for TGFβi NK cell cytokine hypersecretion. To this end, primary NK cells were cultured with IL-2 alone or IL-2 plus TGFβ. In contrast to the effect of TGFβ in combination with the K562 feeder cells, IL-2 plus TGFβ alone did not enhance the anti-tumor IFNγ and TNFα production, in agreement with what has been previously reported (Figure 2B).

Since both K562mbIL-15 and K562mbIL-21 feeder cells express 4-1BBL, we wanted to determine if 4-1BBL was required for TGFβi NK cell cytokine hypersecretion. To determine this, primary NK cells were cultured with irradiated parental K562 in combination with IL-2 and TGFβ, as previously described. NK cells cultured with IL-2 plus TGFβ and parental K562 also exhibited significantly increased IFNγ (Figure 2C) and TNFα (Figure 2C) secretion against MG63 compared to the NK cells expanded on parental K562 without TGFβ. Thus, the expression of membrane-bound IL-21, IL-15, or 4-1BBL on the feeder cells are not required for TGFβ induced cytokine hyperproduction; however, NK cells cultured with parental K562 produced less IFNγ and TNFα in response to MG63 than NK cells cultured with K562mbIL-21, suggesting that membrane-bound IL-21 and 4-1BBL increase the overall cytokine production (Figure S3).

Next, we wanted to determine if cytokine hyperproduction could be induced by TGFβ in the absence of tumor stimulation. IL-12/IL-15/IL-18 has previously been reported to promote the activation of NK cells in the absence of tumor stimulation [12,19]. Thus, primary NK cells were stimulated overnight with IL-12/IL-15/IL-18 plus or minus IL-2 and TGFβ. Following overnight stimulation, the NK cells were washed with saline and continued in culture with IL-15 alone, IL-15 plus TGFβ, IL-15 plus IL-2, or IL-15 plus both IL-2 and TGFβ. IL-2 was used in addition to IL-15 since IL-15 plus TGFβ was not sufficient to induce TGFβi NK cells. A significant increase in IFNγ and TNFα was only observed when IL-2, IL-15, and TGFβ were present (Figure 2D) after the initial cytokine activation, suggesting that an initial activation signal (either cytokine or tumor-induced) and a prolonged culture with IL-2 are required for TGFβ to induce cytokine hyperproduction.

### 2.3. TGFβi generates NK Cells with Increased Degranulation but Transiently Impairs Cytotoxicity

**Figure 3:**
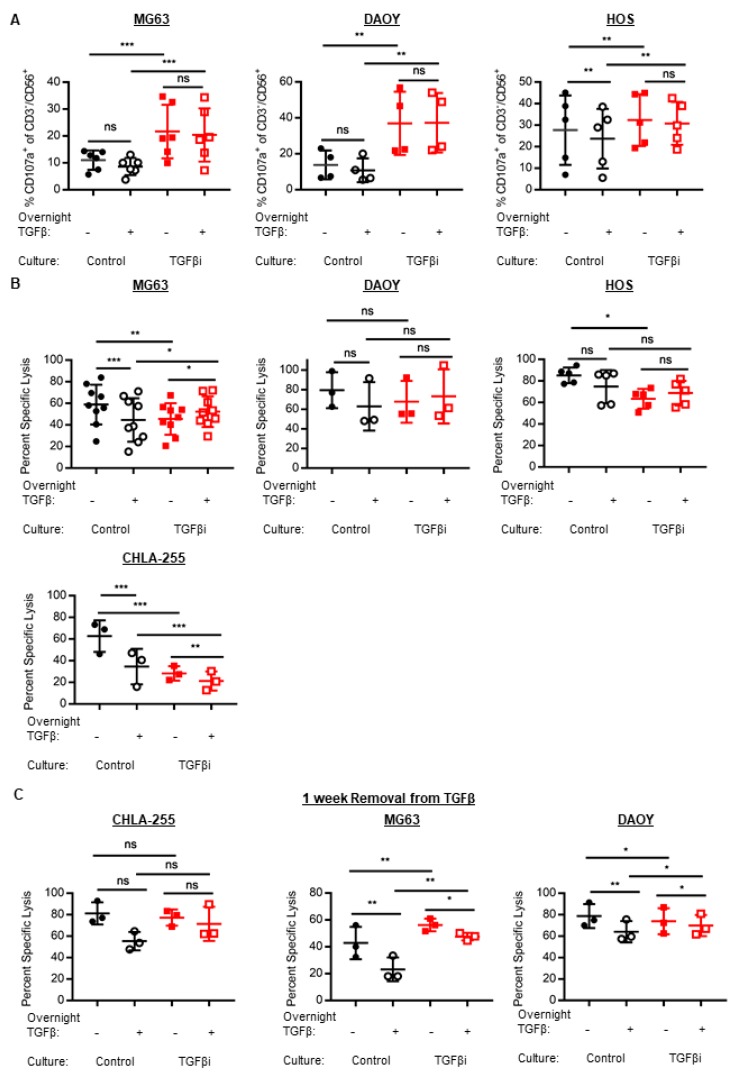
TGFβi generates NK cells with increased degranulation but transiently impairs cytotoxicity. (**A**) Control and TGFβi NK cells from K562mbIL-21 expansions were rested overnight in IL-2 or IL-2 + TGFβ and subsequently stimulated with tumor targets for 3 h (MG63, *n* = 6; DAOY, *n* = 4; HOS, *n* = 5) and assessed for degranulation by CD107a. %CD107a^+^ NK cells are corrected for no target controls. (**B**) The control and TGFβi NK cell cytotoxicity was measured using a 4-h calcein-release cytotoxicity assay, following overnight treatment in IL-2 alone or IL-2 plus TGFβ. (MG63, *n* = 9; DAOY, *n* = 3; HOS, *n* = 5; CHLA-255, *n* = 3). (**C**) TGFβi NK cells were removed from TGFβ for 7 days (±1 day) and cytotoxicity against CHLA-255, MG63, and DAOY following overnight treatment with IL-2 or IL-2 plus TGFβ was measured using a calcein-release assay (*n* = 3). Individual data points depicted for all. Lines and bars represent Mean ± SD. Statistical differences were determined by two-way repeated measures ANOVA with Holm-Sidak’s multiple comparisons test. * *p* ≤ 0.05, ** *p* ≤ 0.01, *** *p* ≤ 0.001, **** *p* ≤ 0.0001.

Since TGFβ potently inhibits NK cell anti-tumor cytotoxicity, we assessed the cytolytic capacity of TGFβi NK cells. TGFβi NK cells had significantly increased anti-tumor degranulation as measured by their surface expression of CD107a against MG63, HOS (osteosarcoma), and DAOY. Degranulation was significantly increased at both the baseline and following overnight acute TGFβ treatment (Figure 3A). Further, although acute overnight TGFβ treatment inhibited the degranulation of control NK cells against HOS, TGFβi NK cells were not inhibited by the acute TGFβ treatment.

However, since degranulation does not always positively correlate with tumor cell death, we measured the ability of TGFβi NK cells to induce tumor cell death using a 4-h calcein release cytotoxicity assay. Against DAOY, the TGFβi NK cells had similar cytotoxicity as control NK cells at both the baseline and following acute TGFβ treatment (Figure 3B). Further, neither the control nor TGFβi NK cytotoxicity was suppressed by acute TGFβ treatment. However, against the osteosarcoma cell lines MG63 and HOS, TGFβi NK cells were significantly inhibited at baseline, but not following the acute TGFβ treatment. In addition, TGFβi NK cells were not suppressed by acute TGFβ; whereas the control NK cell cytotoxicity against MG63 was inhibited by acute TGFβ treatment. The cytotoxicity of TGFβi NK cells against CHLA-255 was markedly inhibited at both baseline and following acute TGFβ treatment. Further, both control and TGFβi NK cells had suppressed cytotoxicity by acute TGFβ treatment. Therefore, the effect of TGFβi on NK cell cytotoxicity is largely cell-line dependent.

Next, we sought to determine if TGFβi NK cells would recover their cytotoxicity following their removal from TGFβ for 1 week. To this end, TGFβi NK cell cytotoxicity was no longer suppressed against CHLA-255 and MG63, suggesting that a short-term removal from TGFβ is an effective strategy to recover cytolytic function in TGFβi NK cells (Figure 3C). However, TGFβi NK cell cytotoxicity was slightly but significantly inhibited following their removal from TGFβ against DAOY at baseline (without overnight TGFβ treatment); whereas, TGFβi NK cell cytotoxicity with overnight TGFβ treatment was slightly but significantly enhanced compared to the control NK cells.

### 2.4. TGFβi Remodels NK Cell Receptor Expression

**Figure 4:**
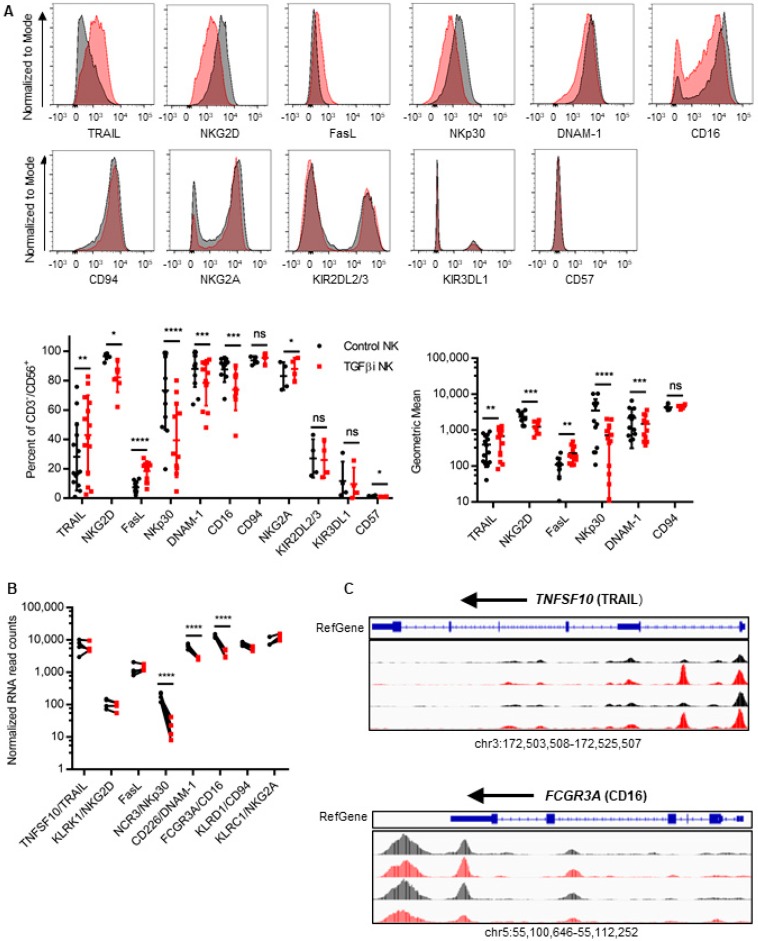
TGFβi remodels K562mbIL-21 expanded NK cell receptor expression. *(***A**) Cell surface protein expression on the control and TGFβi NK cells was measured using flow cytometry. Geometric median fluorescent intensity (gMFI) normalized to viability only stained NK cells are shown. Flow data from one representative donor is depicted. Control in black, TGFβi in red. (**B**) mRNA expression of NK cell receptors was assessed by RNA-seq (*n* = 4). (**C**) Chromatin accessibility of TNSF10 and FCGR3A was determined at Day 14 using ATAC-seq. Control in black, TGFβi in red. Individual Data points depicted. Lines and bars represent Mean ± SD. Statistical differences were determined by paired *t*-test and DESeq2 for RNA-seq. * *p* ≤ 0.05, ** *p* ≤ 0.01, *** *p* ≤ 0.001, **** *p* ≤ 0.0001. See also Figures S4 and S5.

Next, we wanted to determine if TGFβi remodels the NK cell receptor repertoire. To this end, we performed flow cytometry on several NK cell receptors previously reported to be inhibited by acute TGFβ treatment at Day 7, 14, and 21, and confirmed these changes at the transcriptional level at Day 14 using RNA-seq [23,24,25,28,31,32]. We found that TGFβi significantly suppressed NKG2D, CD16, and NKp30 expression and increased FasL and NKG2A expression as early as Day 7 of expansion (Figure 4A and Figure S4A). Conversely, DNAM-1, TRAIL, and CD57 were not significantly affected until after 14 days of expansion. NKp30, DNAM-1, and FCGR3A/CD16 protein expressions correlated with decreased mRNA expression at Day 14 (Figure 4B). We found slight changes in TRAIL and CD16 chromatin accessibility at Day 14 (Figure 4C).

Following one week of removal from TGFβ (Day 21), DNAM-1, FasL and TRAIL expression recovered to similar levels of the control NK cells, whereas CD16 and NKp30 remain suppressed (Figure S4B). It is important to note that the impact of TGFβi on TRAIL expression was highly donor-dependent as 4/6 donors phenotyped at Day 7 did not have changes in TRAIL expression at any time point, despite significant changes in 10/14 donors measured at Day 14. Intriguingly, NKG2D was expressed at significantly higher levels (gMFI) than the control NK cells following one-week removal from TGFβ. Therefore, TGFβi modifies NK cell activating receptor expression.

### 2.5. TGFβi Decreases Granzyme A and Perforin Expression.

**Figure 5:**
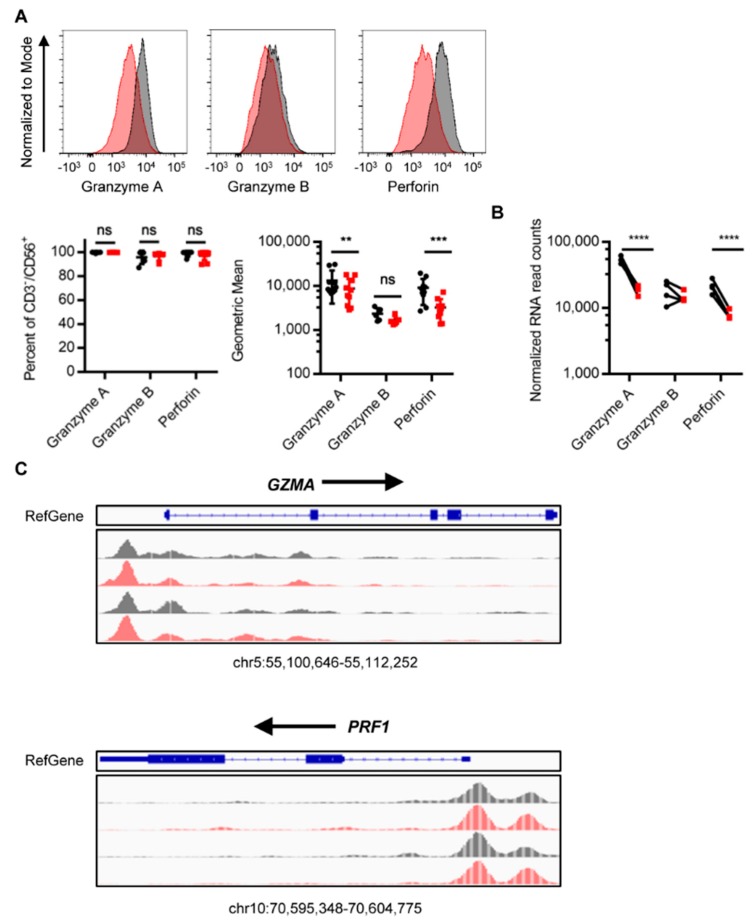
TGFβi decreases granzyme A and perforin expression. (**A**) Granzyme and perforin protein expression in TGFβi NK cells were measured by flow cytometry. Percent positive NK cells and relative expression (gMFI) were depicted. Granzyme A and perforin (*n* = 10), granzyme B (*n* = 5). A representative flow plot is depicted. (**B**) mRNA (*n* = 4 for RNA) of granzymes and perforin was measured using RNA-seq. (*n* = 4 for RNA). (**C**) Chromatin accessibility of GZMA and PRF1 loci as measured by ATAC-seq. Control in black, TGFβi in red. Data are Mean ± SD. Statistical differences were determined by paired *t*-test and RNA-seq by DESeq2. * *p* ≤ 0.05, ** *p* ≤ 0.01, *** *p* ≤ 0.001, **** *p* ≤ 0.0001. See also Figure S5.

Since NK cell granzyme and perforin content can influence NK cell cytotoxicity and the calcein-release cytotoxicity assay is known to predominantly measure granzyme-perforin cytotoxicity, we measured granzyme and perforin expression to determine if their expression could explain the decreased cytotoxicity of TGFβi NK cells against MG63, HOS, and CHLA-255. TGFβi NK cells had a significant reduction in granzyme A and perforin protein (Figure 5A) and mRNA (Figure 5B) (MFI), but no significant change in the percentage of NK cells positive for granzyme A or perforin (Figure 5A). Surprisingly, no significant change was observed in granzyme B mRNA or protein. Next, we wanted to determine if the decrease in granzyme A and perforin expression was correlated with changes in chromatin accessibility. However, there was no significant change in the chromatin accessibility of granzyme A, granzyme B, or perforin in the TGFβi NK cells (Figure 5C and Figure S5). The decrease in cytotoxicity of TGFβi NK cells against some cell lines may also be due to defects in the granzyme-perforin release.

### 2.6. TGFβ Imprinting Modifies NK Cell IFNγ Regulation

**Figure 6:**
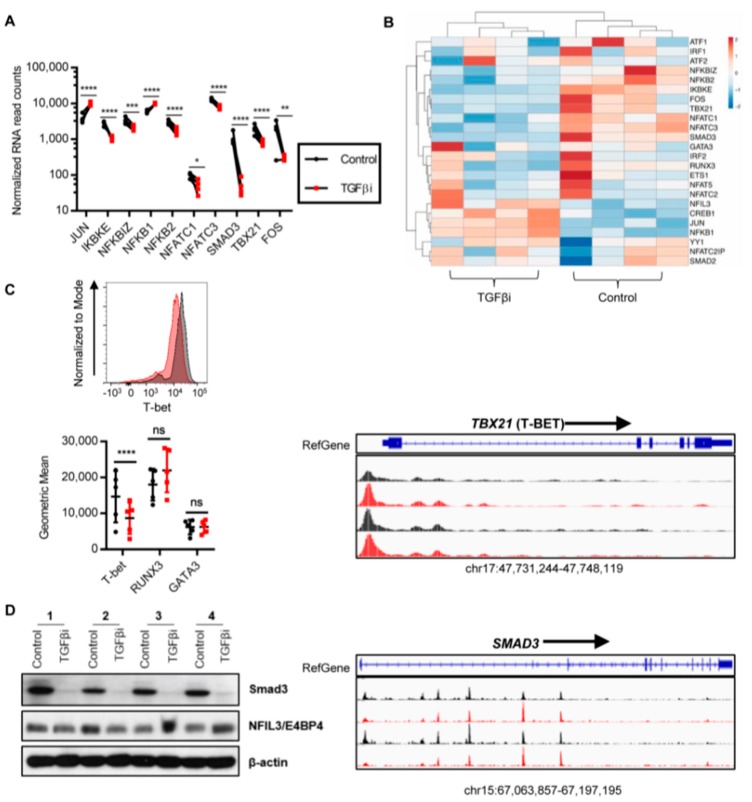
TGFβ imprinting modifies the NK cell IFNγ regulation. See also Figure S4. (**A**) RNA expression of significantly changed IFNγ regulatory genes as assessed using RNA-seq (all *n* = 4). (**B**) Expression of IFNγ regulatory genes shown using Clustvis. (**C**) T-bet protein expression level (gMFI) was assessed using flow cytometry. Representative donor depicted (*n* = 5). Chromatin accessibility of TBX21 (T-bet) was determined using ATAC-seq. Control in black, TGFβi in red. (**D**) SMAD3 and E4BP4 (*n* = 4) protein expressions were measured by western blot. Chromatin accessibility of SMAD3 was measured by ATAC-seq. See also Figure S6. Statistical differences were determined using DESeq2 for RNA-seq and paired *t*-test for flow cytometry. * *p* ≤ 0.05, ** *p* ≤ 0.01, *** *p* ≤ 0.001, **** *p* ≤ 0.0001.

Since TGFβi NK cells have remarkably increased anti-tumor IFNγ secretion, we hypothesized that TGFβ imprinting modified the transcription factors important for IFNγ secretion. To this end, we performed a genome-wide unbiased screening using RNA-seq to determine the expression levels for a broad range of transcription factors. TGFβi NK cells had significantly increased *JUN* and *NFKB1* and significantly decreased *IKBKE*, *NFKBIZ*, *NFKB2*, *NFATC1*, *NFATC3*, *FOS*, *SMAD3*, and *TBX21* (Figure 6A,B). No significant change was observed in *YY1*, *IRF1*, *IRF2*, *NFAT5*, *NFATC2*, *NFATC2IP*, *CREB1*, *ATF1*, *ATF2*, *ETS1*, *RUNX3*, *GATA3*, *NFIL3*, or *SMAD2* (Figure 6B). To further validate that the downregulation of *SMAD3* was a unique alteration in the TGFβ pathway induced by TGFβi, we performed qPCR of 92 genes in the TGFβ pathway and verified their significance against our RNA-seq data. Using this approach, *SMAD3* was 1 of 3 genes significantly altered in the TGFβ pathway, with *TGFBR3* and *SMAD6* also being significantly changed, demonstrating that TGFβi induces selective changes in the TGFβ pathway. Since *SMAD3*, *TBX21* (T-bet), and *NFIL3* (E4BP4) have been demonstrated to be targets of TGFβ signaling that suppress NK cell IFNγ production, we further validated these findings at the protein level. Concordant with the mRNA data, we found that SMAD3 and T-bet were decreased at the protein level while E4BP4 was not significantly changed (Figure 6C,D). In addition, *SMAD3* chromatin accessibility was decreased in TGFβi NK cells suggesting epigenetic remodulation as the mechanism of SMAD3 downregulation. In contrast, *TBX21* (T-bet) chromatin was not remodeled suggesting other mechanisms of mRNA downregulation (Figure 6C,D).

Next, we sought to determine the onset and duration of SMAD3 and T-bet suppression following TGFβi. To this end, we assessed the expression of SMAD3 and T-bet in the control and TGFβi NK cells at Day 7 and Day 14 of the expansion and 1 week following their removal from TGFβ at Day 21 (Figure S4C). SMAD3 suppression was observed at Day 7, and the expression of SMAD3 was recovering at Day 21. Similarly, the T-bet expression also recovered by Day 21. Thus, in contrast to previous studies, our results suggest that when chronically exposed in the presence of IL-2, TGFβ does not inhibit the E4BP4 expression and that TGFβi NK cell IFNγ hypersecretion does not correlate with T-bet expression.

## 3. Discussion

Numerous studies have demonstrated that TGFβ is a potent suppressor of NK cell anti-tumor activity by inhibiting cytotoxicity, cytokine secretion, and NK cell proliferation. Herein, our data demonstrate for the first time that the activation of NK cells in the presence of IL-2 and TGFβ drives NK cells into a pro-inflammatory phenotype with hypersecretion of IFNγ, TNFα, and GM-CSF. This cytokine hypersecretion persists for at least one month following TGFβ imprinting and corresponds to changes in several IFNγ regulatory genes including *JUN*, T-bet, and SMAD3 [19]. Further, TGFβi NK cells have altered cytotoxicity that is cell-line dependent (increased in some and decreased in others), likely due to decreased granzyme A and perforin expression. Therefore, the chronic co-stimulation of NK cells with IL-2 and TGFβ during cytokine and tumor activation represents a novel setting in which TGFβ induces pro-inflammatory NK cells.

Our data demonstrate that chronic TGFβ imprinting in conjunction with activation and IL-2 has opposite effects of acute TGFβ exposure (6 h to 3 days), driving NK cells to produce more IFNγ and TNFα upon tumor target and PHA stimulation than NK cells that were not TGFβ imprinted [18,19,21,31]. Further, TGFβi selectively modulated NK cell cytokine secretion increasing IFNγ, TNFα, and GM-CSF secretion, but not IFNα, IL-2, IL-4, IL-5, IL-10, IL-12, or IL-17A. Interestingly, TGFβi NK cell cytokine hypersecretion could be induced by both feeder cell stimulation and, in the absence of feeder cell stimulation, by using IL-2 plus IL-12/IL-15/IL-18 with TGFβ supplementation. Thus, TGFβi NK cell cytokine hypersecretion develops independently of the receptor-ligand interactions between NK cells and K562 feeder cells, and likely represents a common activation pathway shared between cytokine and tumor-stimulated NK cells. In addition, cytokine secretion was not dependent upon the receptor-ligand interactions between tumor targets and NK cells, rather, the increased cytokine secretion following PHA stimulation suggests that TGFβi cytokine hypersecretion likely reflects transcriptional and translational changes. Furthermore, these changes persist following the recovery of SMAD3, T-bet, and NKG2D expression.

Whether TGFβi NK cells develop naturally in vivo remains to be determined. There is potential for TGFβi to occur during chronic activation in the body such as in the tumor microenvironment or during wound healing. However, since TGFβi requires extended TGFβ exposure, NK cells must remain in these pro-inflammatory microenvironments for several days to induce TGFβi. Despite following the phenotypic changes of TGFβi NK cells during expansion and following the removal from TGFβ, we were unable to identify a cell-surface marker that correlated with the acquisition of cytokine hypersecretion (Day 14 in K562mbIL-21 expanded NK cells). The identification of a cell-surface marker of TGFβi to identify these NK cells in vivo is an important area of future investigation.

A positive effect of TGFβ on NK cell function was suggested in a recent study demonstrating that TGFβ signaling increases NK cell anti-tumor activity, as SMAD4^−/−^ murine NK cells had decreased cytotoxicity [33]. In addition, TGFβ is necessary for the development of salivary gland ILC1s and was able to convert NK cells into ILC1s [34,35]. Further, TGFβ promotes survival of salivary gland ILC1s and some T-cell subsets, raising the question of whether TGFβ is similarly promoting the survival of TGFβi NK cells [34]. This may be enhancing cytokine and tumor activation such as by IL-2 and K562 feeder cells, thereby alleviating cytokine-induced death [34,36,37,38]. Similar to our TGFβi NK cells, TRAIL expression was upregulated on the salivary gland ILC1s by TGFβ. However, ILC1s were not potent IFNγ producers and conversion of NK cells into ILC1s was associated with decreased anti-tumor activity [34,35]. In contrast, TGFβi NK cells maintain anti-tumor cytotoxicity against some cell lines (DAOY) and produce more IFNγ than NK cells cultured without TGFβ, suggesting that TGFβ in this system does not convert NK cells into ILC1s.

In agreement with previous studies, both acute TGFβ-treated NK cells and chronic TGFβi NK cells had decreased cytotoxicity against some cell lines [21,25,26,31,35]. Despite decreased activating receptor expression, degranulation by TGFβi NK cells was not inhibited, suggesting no defect in the activation by tumor targets; however, degranulation did not correlate with cytotoxicity. This effect is likely due to the decreased granule content in TGFβi NK cells, as the effectiveness of degranulation in mediating cell death is largely dependent upon the granzyme/perforin content [39]. The cell-line dependent effect of TGFβi on NK cell cytotoxicity likely represents different pathways of cytotoxicity which will be of particular interest for future investigations. Further, it is intriguing that TGFβi NK cells recover their cytotoxicity against CHLA-255 and MG63 following their removal from TGFβ while maintaining TGFβ-induced pro-inflammatory cytokine secretion, suggesting that TGFβ has effects on cytotoxicity that are not epigenetic. Cytotoxicity recovery may be due to the recovery of NKG2D and DNAM-1 expression following their removal from TGFβ.

TGFβi NK cells have a differential expression of several transcription factors known to be important for NK cell IFNγ secretion. Previously, it was reported that both T-bet and E4BP4 were suppressed by TGFβ, leading to a decrease in IFNγ secretion. Similarly, we found that T-bet decreased following TGFβ in our TGFβi NK cells; however, surprisingly, TGFβi NK cells produced significantly more IFNγ. Thus, the residual T-bet expression may be able to promote IFNγ expression more efficiently in the absence of SMAD3, or other transcription factors may promote IFNγ secretion in this setting. TGFβi NK cells had similar levels of E4BP4, which may be due to the loss of SMAD3 in TGFβi NK cells since SMAD3 has been reported to suppress E4BP4 expression [20]. In contrast, TGFβ can suppress T-bet expression through SMAD2 in the absence of SMAD3. Thus, SMAD2 may be downregulating T-bet expression in TGFβi NK cells [19]. We found increased *Jun* and *NFKB1* which are known to be important in activation-induced transcription of IFNγ, suggesting that they may be upregulating IFNγ transcription in TGFβi NK cells [40,41,42].

Based on their enhanced cytokine production and their TRAIL and FasL expression, TGFβi NK cells may be an effective novel therapy in certain solid tumors. TGFβi NK may have increased efficacy in vivo in settings where IFNγ can activate macrophages and CD8 T cells to stimulate adaptive immunity. Additionally, IFNγ itself can sensitize tumors to NK cell cytotoxicity [43,44,45,46,47,48]. In addition, TNFα can directly kill tumors expressing TNFRs and works in concert with IFNγ to inhibit cancer cell proliferation [47]. Further, FasL and TRAIL may be preferentially used by NK cells during in vivo cytotoxicity compared to in vitro cytotoxicity [49,50]. Thus, future studies are needed to determine the therapeutic potential of TGFβi NK.

## 4. Conclusions

In summary, TGFβ’s effect on NK cells is context-dependent, such that chronic TGFβ in the presence of IL-2 and activating signals generates NK cells that hypersecrete IFNγ, TNFα, and GM-CSF that persists after their removal from TGFβ. In contrast, TGFβi transiently impairs in vitro cytotoxicity, which recovers rapidly following their removal from TGFβ. The data reported here delineates a novel effect where TGFβ can induce pro-inflammatory NK cells.

## 5. Materials and Methods 

### 5.1. Cell Lines and Culture Conditions

Experiments using discarded anonymized buffy coats from normal human red blood cell (RBC) donations from the American Red Cross (Columbus, OH, USA) were IRB exempt. Blood was processed using Ficoll Plus (GE Healthcare; 17-1440-02), as described previously. Human NK cells were purified with a RosetteSep Human NK Cell Enrichment Cocktail (Stem Cell Technologies, 15065, Vancouver, BC, Canada) as described in Reference [51]. NK cell purity was greater than 85%, with less than 5% contaminating T-cells.

Cell line identity was authenticated using STR fingerprinting (Idexx Biosciences, Columbia, MO, USA) and routinely tested for mycoplasma contamination. DAOY was cultured in MEM with 1% Non-essential amino acids, 1% glutamax, 1% P/S and 10% FBS. MG63 and HOS were cultured in DMEM with 1% P/S and 10% FBS. CHLA-255 was cultured in 20% FBS in IMDM with 1% glutamax, 1% ITS, and 1% P/S. Cells were dissociated for cytotoxicity assays using an enzyme-free Cell Dissociation Buffer, Hank’s Based (ThermoFisher Scientific, 13150016, Carlsbad, CA, USA). NK cells were cultured in RPMI 1640 media plus Glutamax, 10% FBS, and P/S. K562 feeder cells were derived by transducing K562, a chronic myelogenous leukemia cell line, with human 4-1BBL and membrane-bound human IL-21, as previously described [11].

Purified primary human NK cells were stimulated at Day 0 1:2 with 100 Gy irradiated K562 mbIL-21, K562mbIL-15, or parental K562 as indicated and 1:1 at Day 7. Control expanded NK cells were supplemented with 50 IU/mL recombinant human IL-2, and TGFβi expanded NK cells received 50 IU/mL IL-2 (Prometheus, 22mmu NDC 65483-0116-07, San Diego, CA, USA) plus 10 ng/mL TGFβ (Biolegend, 580706, San Diego, CA, USA) every 2–3 days. Day 14 NK cell purity was ≥90% CD3^−^/CD56^+^, and CD3^−^/CD16^+^ cells. NK Cell Expansion was calculated based on the percentage of CD3^−^/CD56^+^/CD16^+/−^ cells within the lymphocyte gate as determined by FSC/SSC.

For measuring the persistence of TGFβi NK cell function, donor-matched NK cells were expanded as described above using K562 mbIL-21 feeder cells. After 14 days, the NK cells were rested in 50 IU/mL IL-2 alone for 7–33 days and IFNγ and TNFα secretion was measured at a 5:1 (NK: tumor) ratio following 3 h co-culture with DAOY using BD Cytometric Bead Array (details of this method are described below).

For NK cell stimulation with IL-12, IL-15, and IL-18, primary NK cells were stimulated overnight with 10 ng/mL IL-12 (Biolegend, 573002), 50 ng/mL IL-15 (Biolegend, 570302) and 50 ng/mL IL-18 (Biolegend, 592102) as described and rested in 1 ng/mL IL-15 for 7–14 days following overnight stimulation with IL-12, IL-15, and IL-18 [12]. For determining the effect of IL-2 and TGFβ on cytokine production, the NK cells were treated as described but with the addition of IL-2 and/or TGFβ as indicated in the overnight stimulation with IL-12, IL-15, and IL-18, and along with 1 ng/mL IL-15 for 7–14 days. To measure the cytokine production, the NK cells were rested in 1 ng/mL IL-15 only overnight and throughout the assay and co-cultured with MG63 at a 5:1 ratio or equal numbers of NK cells only as a no target control and intracellular flow staining was conducted as described below.

### 5.2. Flow Cytometry

The staining of human NK cells was conducted as described previously in blocking buffer of 50% FBS/PBS. Transcription factors were stained using the Transcription Factor Buffer Kit (BD Biosciences, 562725, San Diego, CA, USA). All other intracellular flow cytometry studies were done using the BD Cytofix/Cytoperm Fixation/Permeabilization Kit with GolgiStop (BD Biosciences, 554715). Antibodies for the following proteins were used to assess NK phenotype and function: CD3 PeCy7/APC-H7 (BD Biosciences, clone SK7), CD56 FITC/BB515/BV421 (BD Biosciences, Clone NCAM16.2; clone B159, clone R19-760)), NKG2D Pe-CF594/BV510 (BD Biosciences, clone 1D11), TRAIL PE/APC/BV421 (BD Biosciences, clone RIK-2), FasL PE (BD Biosciences, clone NOK-1), NKp30 PE/Alexa Fluor 647 (BD Biosciences, Cat#: 558407, 558408), NKp30 PE-Vio615 (Miltenyi, Cat#: 130-112-434, Bergisch Gladbach, Germany), CD94 APC (Miltenyi, Cat#: 130-098-976), CD57 Pe-Vio615 (Miltenyi, Cat#: 130-111-815), KIR3DL1 BV421 (BD Biosciences, clone DX9), KIR2DL2/3 Pe-Vio770 (Miltenyi, Cat#: 130-099-892), NKG2A APC (Miltenyi, Cat#: 130-098-812), granzyme A APC (Miltenyi, Cat#: 130-099-780), granzyme B BV510 (BD Biosciences, clone GB11), perforin BV421 (BD Biosciences, clone δG9), DNAM-1 BV711 (BD Biosciences, clone DX11), CD107a BV510 (BD Biosciences, clone H4A3), IFNγ APC (Biolegend, clone 4S.B3), TNFα BV421 (Biolegend, clone Mab11), CD16 PE/APC (BD Biosciences, clone B73.1; Miltenyi, Cat#: 130-106-705)), T-bet APC (Biolegend, clone 4B10), GATA3 BV421 (Biolegend, clone 16E10A23), RUNX3 PE (BD Pharmingen, R3-5G4, San Diego, CA, USA), and Tonbo Ghost Dye 510/710/780 (Tonbo Biosciences, San Diego, CA, USA). For determining the percent of NK cells expressing proteins, cells were gated on Live/CD3^−^/CD56^+^ for all analysis.

To determine degranulation by CD107a expression and intracellular cytokine production in response to tumors, NK cells were rested overnight in fresh media containing either 50 IU/mL of IL-2 (baseline) or 50 IU/mL IL-2 and 10 ng/mL TGFβ. Following overnight cytokine stimulation, the NK cells were resuspended in fresh media with the same cytokines as used in the overnight cytokine stimulation. The NK cells were co-cultured in a 96-well round-bottom plate with tumor cells (5:1 E:T ratio) or no target for a control in 200 µL media as described for cytotoxicity assays. One µL of monensin was added to each sample along with CD107a at the beginning of the assay. Plates were spun down at 100 g × 2 min to promote cell-cell contact and placed in a 37 °C incubator for 3 h (NK cells expanded with K562mbIL-21, K562mbIL-15, and K562 parental) or 6 h (cytokine only stimulations). After incubation, the media were removed and staining began for cell surface and intracellular proteins as detailed. To determine tumor-specific NK cell production, CD107a, IFNγ, and TNFα percent of NK cells and gMFI were normalized to NK cells with no target.

### 5.3. Cytotoxicity Assay

Calcein (Invitrogen, C3099, Carlsbad, CA, USA) assays were conducted as described previously. NK cells were prepared for cytotoxicity assays as described for intracellular functional flow. NK cells and tumors were cultured at a 5:1 Effector:Target ratio.

### 5.4. Cytokine Secretion

To determine the NK cell release of IFNγ and TNFα, NK cells were cultured as described for intracellular functional flow cytometry with the exception of the monensin and CD107a antibody. After 3 h co-culture with tumor targets or 4 h stimulation with 10 µg/mL PHA, supernatants were collected and frozen at −75 °C until use. On the day of the assay, the supernatants were thawed and 50 µL of undiluted supernatant was used according to the manufacturer’s instructions for the BD CBA Soluble Protein Master Kit (BD Biosciences, Cat#: 558265) and IFNγ and TNFα Flex Set (BD Biosciences, Cat: 558269, 560112) or MACSPlex Cytokine 12 Kit (Miltenyi, Cat: 130-099-169). The analytes were acquired on a BD LSR II or a MACSQuant. The geometric mean for each analyte was determined in Flow Jo v. 10.1 and unknown samples were interpolated using a standard curve with R^2^ ≥ 0.9 from the known standards for BD LSR II acquired samples. Analysis of MACSQuant acquired analytes was done using MACSQuantify software (version 2.8, Bergisch Gladbach, Germany). This software uses average APC median values of MACSPlex Standards and calculates the cytokine concentration in each sample.

### 5.5. qPCR

RNA from fresh, never frozen, Day 14 K562mbIL-21 expanded human NK cells was isolated using the Qiagen MiniPrep kit. cDNA was made using the high throughput cDNA kit (Applied Biosystems, #4368814, Carlsbad, CA, USA). PCR for the TGFβ pathway was done using a Taqman Fast PCR Mastermix and Human Fast 96-well TGFβ Pathway Array (ThermoFisher, 4418742, Pleasanton, CA, USA) on an Applied Biosystems 7900HT.

### 5.6. RNA-Seq Sample Preparation and Sequencing

Total RNA was prepared from K562 mbIL-21 expanded control and TGFβi NK cells as per manufacturer’s instructions using the Total RNA Purification Plus Kit (Norgen Biotek, Thorold, ON, Canada) and the resulting total RNA was quantified in a Nanodrop ND-1000 spectrophotometer, and checked for purity and integrity in a Bioanalyzer-2100 device (Agilent Technologies Inc., Santa Clara, CA, USA) and submitted to the genomics core at the Nationwide Children’s Hospital for sequencing. Libraries were prepared using the TruSeq RNA Sample Preparation Kit (Illumina Inc., San Diego, CA, USA) according to the protocols recommended by the manufacturer. The quality of the libraries were determined via Agilent 4200 Tapestation using a High Sensitivity D1000 ScreenTape Assay kit and quantified by KAPA qPCR (KAPA BioSystems, Cape Town, South Africa). Approximately 60–80 million paired-end 150 bp sequence reads per library were generated using the Illumina HiSeq4000 platform.

### 5.7. ATAC-seq

K562mbIL-21 expanded control and TGFβi NK cells were counted, resuspended in growth media containing 5% DMSO and then aliquoted in cryovials containing 100,000 viable cells/vial. Cells were frozen at a slow cooling rate and stored at −80 °C prior to processing for ATAC-seq. ATAC-seq was performed as described [52]. DNA libraries were sequenced using Illumina HiSeq 2500 at 50 bp paired end reads.

### 5.8. Western Blotting

Whole cell extracts were isolated from K562mbIL-21 expanded NK cells using a RIPA Lysis Buffer (Pierce Biotechnology, Rockford, IL, USA) supplemented with protease and phosphatase inhibitors (Thermo Scientific). The concentration of the isolated proteins was determined using a BSA Protein Assay kit (Bio-Rad, Santa Rosa, CA, USA). Fifteen-twenty micrograms of the protein were separated on SDS-PAGE or 4–12% NuPage Bis-Tris gel (Thermo Scientific) and electrophoretically transferred to PVDF membranes. Membranes were then incubated with the primary antibodies against the proteins of interest Smad3 (Clone C67H9), NFIL3 (clone D5K8O), T-bet (clone D6N8B), or β-actin (clone 8H10D10) (all Cell Signaling, Cambridge, MA, USA) and were detected with HRP-conjugated appropriate secondary antibodies either anti-rabbit IgG, HRP-linked (Cell Signaling, Cat: 7074S) or anti-mouse IgG, HRP-linked (Cell Signaling, Cat: 7076S) and visualized with the ECL Western blotting substrate (Pierce Biotechnology, Rockford, IL, USA), according to the provided protocol.

### 5.9. Statistical Analysis

Statistical analyses were performed as described in each figure legend using GraphPad Prism 6.0 or 7.0 (La Jolla, CA, USA). *p* Values less than 0.05 were considered significant. The heatmap and unsupervised clustering were analyzed using Clustvis [53].

## 6. Patents

D.A.L., J.A.F. and J.E.M. have submitted a US patent on intellectual property relevant to this manuscript.

## Supplementary Materials

The following are available online at http://www.mdpi.com/2072-6694/10/11/423/s1, Figure S1: TGFβi NK cells have increased anti-tumor IFNγ+ and TNFα+ NK cells; Figure S2: TGFβ imprinting increases intensity of IFNγ and TNFα production; Figure S3. Differential IFNγ and TNFα secretion induced by parental K562 versus K562mbIL-21 feeder cell expansion; Figure S4. Related to Figure 4 and Figure 6. Phenotypic analysis of TGFβi NK cells at Day 7, 14, & 21; Figure S5: ATAC-seq results for TGFβ imprinted NK cell phenotype; Figure S6: ATAC-seq results for TGFβ imprinted NK cell transcription factors.
