# Supplementary material for: TGFβ Imprinting During Activation Promotes Natural Killer Cell Cytokine Hypersecretion
Source: Cancers (Basel). 2018 Nov 5;10(11):423. doi: 10.3390/cancers10110423 (PMC6267005; doi:10.3390/cancers10110423)
Supplement: Supplementary file 1 [file cancers-10-00423-s001.pdf]

cancers

MDPI

Supplementary Materials: TGFβ Imprinting During Activation Promotes Natural Killer Cell Cytokine Hypersecretion

Jennifer A. Foltz, Jena E. Moseman, Aarohi Thakkar, Nitin Chakravarti and Dean A. Lee

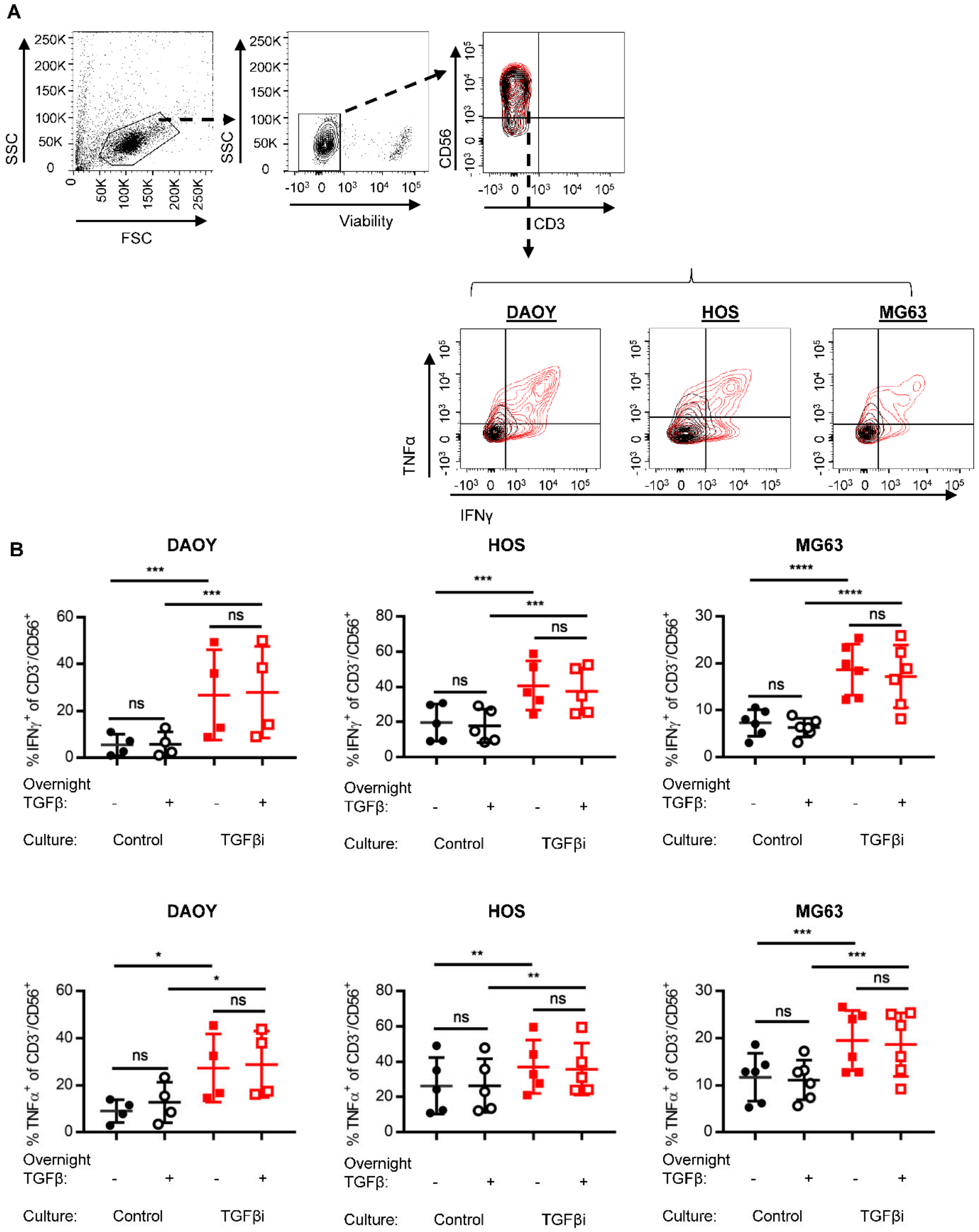

Figure S1. Related to Figure 1. TGFβi NK cells have increased anti-tumor IFNγ+ and TNFα+ NK cells. (**A**) Representative flow gating depicting IFNγ+ and TNFα+ against DAOY, HOS, and MG63. Control in black, TGFβi in red. (**A**) TGFβi increases anti-tumor IFNγ+ and (**B**) TNFα+ at baseline and in the

presence of additional TGFβ. Individual data points depicted. Lines and Bars are Mean ± SD. Statistical differences were determined by two-way repeated measures ANOVA with Holm-Sidak's multiple comparisons test. * $p \leq 0.05$, ** $p \leq 0.01$, *** $p \leq 0.001$, **** $p \leq 0.0001$.

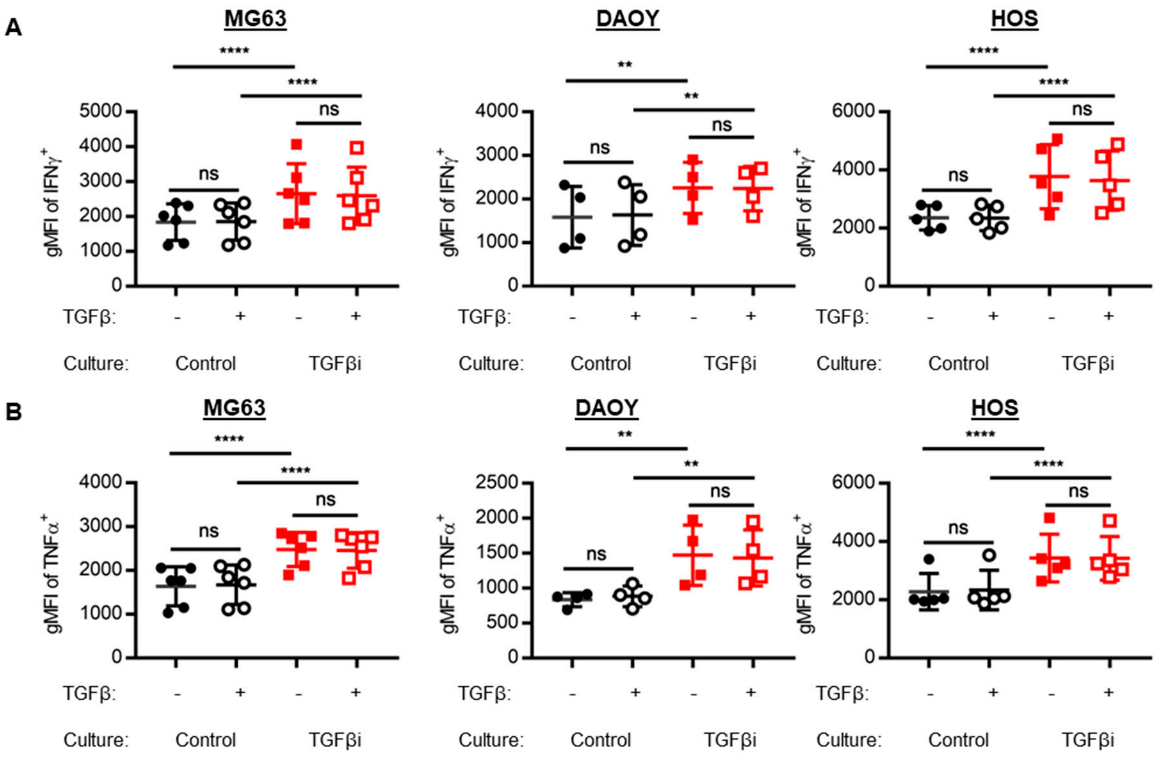

Figure S2. Related to Figure 1. TGFβ imprinting increases intensity of IFNγ and TNFα production. (**A**) TGFβi NK cells have increased geometric mean (gMFI) of IFNγ and (**B**) TNFα. Individual data points depicted. Lines and bars represent Mean ± SD. Statistical differences were determined by two-way repeated measures ANOVA with Holm-Sidak's multiple comparisons test. * $p \leq 0.05$, ** $p \leq 0.01$, *** $p \leq 0.001$, **** $p \leq 0.0001$.

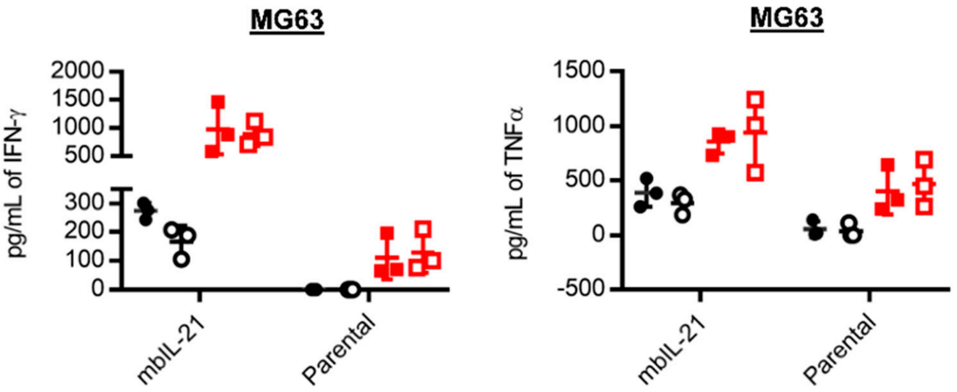

Figure S3. Differential IFNγ and TNFα secretion induced by parental K562 versus K562mbIL-21 feeder cell expansion. NK cells were expanded from identical donors for 2 weeks on either parental K562 or K562mbIL-21 plus IL-2 (Control, black) or IL-2 plus TGFβ (TGFβi, red). At the end of 2 weeks, NK cells were rested overnight in IL-2 only (solid symbols) or IL-2 plus TGFβ (open symbols) and cultured with MG63 for 3 h. Cytokine secretion was measured in the supernatants using CBA analysis ($n = 3$). Individual data points depicted. Lines and bars represent Mean ± SD.

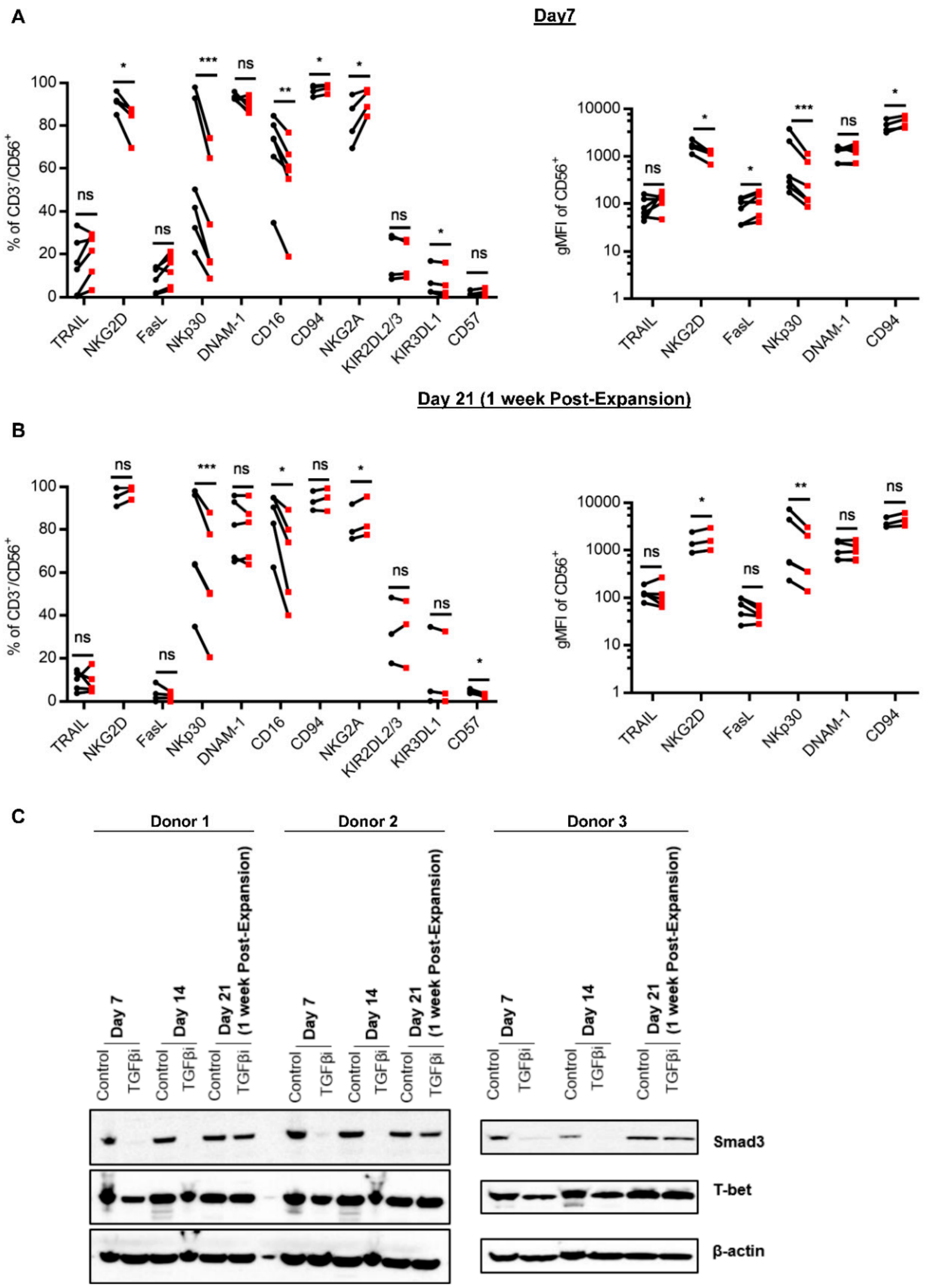

Figure S4. Phenotypic analysis of TGFβi NK cells at Day 7, 14 and 21. Control and TGFβi NK cell receptor repertoire was assessed using flow cytometry at (**A**) Day 7 and (**B**) one week following removal from TGFβ at Day 21 ($n = 3$–6). (**C**) SMAD3 and T-bet expression was similarly measured by western blot at Day 7, Day 14 and Day 21. Individual data points depicted. Lines connect donors. Statistical differences were determined by paired *t*-test. * $p \leq 0.05$, ** $p \leq 0.01$, *** $p \leq 0.001$, **** $p \leq 0.0001$. Related to Figures 4&6.

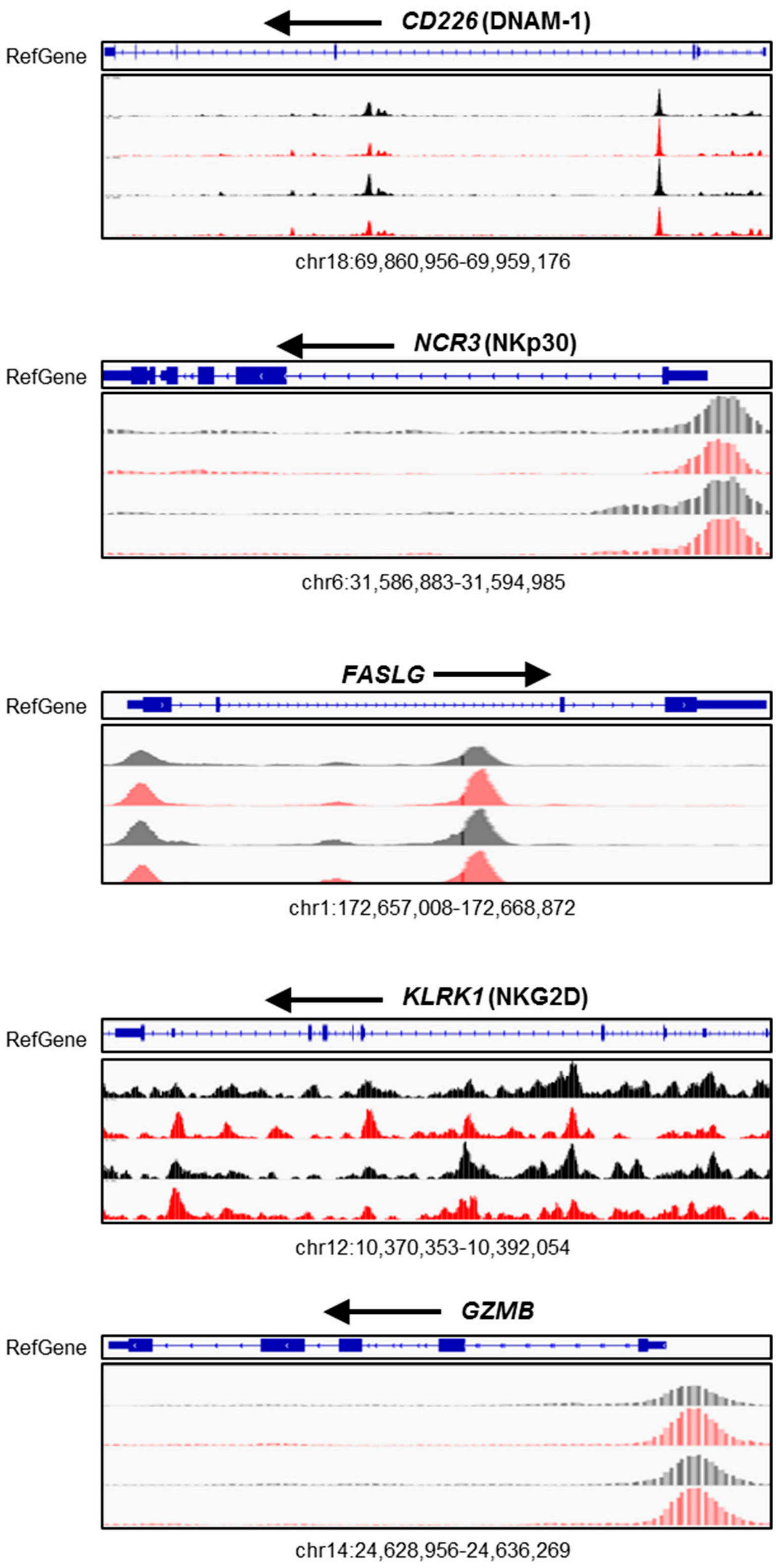

Figure S5. ATAC-seq results for TGFβ imprinted NK cell phenotype. Control in black. TGFβi in red. $n = 2$. Related to Figures 4 & 5.

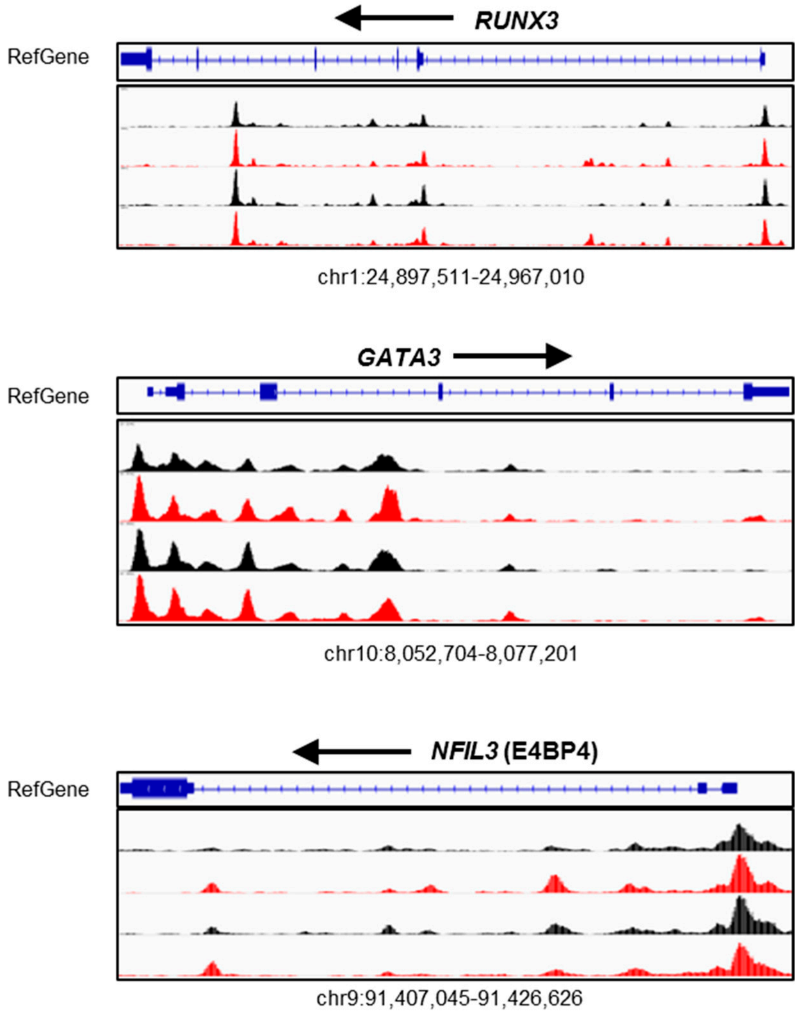

Figure S6. ATAC-seq results for TGFβ imprinted NK cell transcription Factors. Control in black. TGFβi in red. $n = 2$. Related to Figures 6.
